# Dominant Action of *CLCN4* Neurodevelopmental Disease Variants in Heteromeric Endosomal ClC-3/ClC-4 Transporters

**DOI:** 10.3390/cells14241973

**Published:** 2025-12-11

**Authors:** Abraham Tettey-Matey, Alessandra Picollo, Francesca Sbrana, Maria Antonietta Coppola, Eugenia Rubino, Alice Giusto, Margherita Festa, Elena Angeli, Cristiana Picco, Raffaella Barbieri, Paola Gavazzo, Michael Pusch

**Affiliations:** 1 Istituto di Biofisica, Consiglio Nazionale delle Ricerche, 16149 Genova, Italy; abraham.matey@ibf.cnr.it (A.T.-M.); alessandra.picollo@ibf.cnr.it (A.P.); francesca.sbrana@ibf.cnr.it (F.S.); mariaantonietta.coppola@ibf.cnr.it (M.A.C.); eugenia.rubino@ibf.cnr.it (E.R.); alice.giusto@ibf.cnr.it (A.G.); margherita.festa@ibf.cnr.it (M.F.); cristiana.picco@ibf.cnr.it (C.P.); raffaella.barbieri@ibf.cnr.it (R.B.); paola.gavazzo@ibf.cnr.it (P.G.); 2 DIFI Lab, Dipartimento di Fisica, Università di Genova, 16146 Genova, Italy; elena.angeli@unige.it

**Keywords:** neurodevelopmental disorder, endosome, X-linked disease, chloride transport, CLC proteins

## Abstract

**Highlights:**

**What are the main findings?**

- Several *CLCN4* variants exert dominant effects within ClC-3/ClC-4 heterodimers, as demonstrated using electrophysiological assays.
- The study provides the first evidence of dominant behavior of *CLCN4* variants in mixed ClC-3/ClC-4 complexes and introduces a robust platform for assessing additional disease-associated variants.

**What are the implications of the main findings?**

- Dominant effects of *CLCN4* variants may help explain the broad phenotypic variability observed in neurodevelopmental disorders linked to *CLCN4*, including severe presentation in individuals with partial or complete LoF missense variants.
- The new experimental platform enables systematic evaluation of emerging *CLCN4* and *CLCN3* variants, improving genotype–phenotype interpretation and supporting future diagnostic and therapeutic strategies.

**Abstract:**

Variants in *CLCN3* and *CLCN4*, encoding the neuronal endosomal Cl^−^/H^+^ antiporters ClC-3 and ClC-4, are linked to neurodevelopmental disorders with broad phenotypic variability. Over sixty *CLCN4* variants have been functionally characterized, showing gain- or loss-of-function (GoF or LoF) effects. While ClC-3 can function as a homodimer, ClC-4 depends on heterodimerization with ClC-3 for efficient endosomal trafficking. *CLCN4*, located on the X chromosome, exhibits diverse pathogenic outcomes: complete LoF variants often cause non-syndromic presentations in hemizygous males and are asymptomatic in heterozygous females, whereas certain missense variants with partial or complete LoF produce severe syndromic phenotypes in both sexes. Here, we demonstrate dominant effects of three *CLCN4* variants within ClC-3/ClC-4 heterodimers using two-electrode voltage-clamp recordings in Xenopus laevis oocytes and whole-cell patch-clamp recordings in mammalian cells co-expressing both proteins via a bicistronic IRES construct. Our findings provide the first evidence of dominant-negative *CLCN4* effects within ClC-3/ClC-4 complexes and establish a platform for functional analysis of additional disease-associated variants.

## 1. Introduction

Genetically inherited neurodevelopmental disorders (NDDs) represent a diverse range of conditions marked by brain development, leading to deficits in cognition, behavior, and motor skills. Clinical manifestations include intellectual disability, autism spectrum disorder, global developmental delay, and attention-deficit/hyperactivity disorder, often accompanied by additional features such as epilepsy [1]. NDDs affect millions globally and frequently lead to lifelong disability with diverse phenotypic presentations.

Among the genes implicated in NDDs, those encoding endosomal CLC proteins—particularly ClC-3, ClC-4, ClC-6, and ClC-7—play a critical role in brain development. These proteins regulate ion homeostasis within neuronal endosomal-lysosomal compartments. All these vesicular CLCs function as 2Cl^−^/H^+^ exchangers. Such activity can in principle help in acidification of intracellular vesicles such as endosomes and lysosomes by the V-type H^+^ ATPase by providing counter charge [2,3]. However, it has also been proposed that at least some vesicular CLCs are essential for maintaining proper luminal chloride accumulation [4,5]. These processes are crucial for vesicle trafficking, degradation, and autophagy, all of which are vital for neuronal function and survival [4,6,7].

Despite their structural similarity to plasma membrane-localized CLC chloride channels, such as ClC-1, ClC-2, ClC-Ka, and ClC-Kb, vesicular CLC proteins are functionally distinct, underscoring their specialized roles within intracellular compartments [4].

A key feature of all CLC proteins is their homo- (or hetero-) dimeric architecture, with each subunit containing an independent ion transport pathway [4]. Additionally, a “common gate” shared by both subunits can regulate transport, such that the “closure” of the gate blocks ion movement simultaneously in both subunits. The precise molecular nature of this common gate remains unresolved [4].

Variants in both *CLCN3* and *CLCN4* are associated with neurodevelopmental disorders that share partially overlapping but distinct symptom profiles [8,9,10,11,12,13,14]. In particular, patients with variants in *CLCN3* often present with early-onset neurodevelopmental delay, seizures, and moderate-to-severe intellectual disability. Additional clinical features may include hypotonia, movement disorders, visual impairment, and progressive cerebral or cerebellar atrophy on neuroimaging [12,14]. Some individuals also show behavioral abnormalities, feeding difficulties, or peripheral neuropathy. Pathogenic variants in *CLCN4* cause an X-linked neurodevelopmental and epilepsy syndrome marked by global developmental delay, intellectual disability ranging from mild to profound, and a spectrum of seizure types that may be difficult to control. Other common features include autistic behaviors, anxiety or aggression, movement abnormalities (such as ataxia or dystonia), microcephaly, gastrointestinal dysmotility, and variable dysmorphic features [10,11,13,15,16]. In vitro functional analyses of disease-associated variants have revealed both LoF and GoF effects [8,10,11,12,13,15]. Interestingly, a significant proportion of variants display functional profiles indistinguishable from wild-type (WT) [12,13,15].

Some of these WT-like *CLCN4* variants exhibit impaired heteromerization with ClC-3 [15], which may contribute to the observed disease phenotype. A recent breakthrough demonstrating that ClC-3 and ClC-4 strongly interact with the single-span transmembrane proteins TMEM9 and TMEM9B [17,18] raises the possibility that WT-like variants might exert pathogenic effects by disrupting this interaction. Indeed, using a novel vacuolation assay, Planells-Cases and colleagues showed that some variants significantly reduce the inhibitory effect of TMEM9(B) on ClC-3, resulting in a functional GoF [18].

To date, *CLCN4* variants have primarily been studied in the context of homodimeric complexes, which, although informative for understanding biophysical effects, do not fully reflect the physiological conditions. Two key considerations are critical for accurately interpreting the functional consequences of *CLCN4* variants. First, *CLCN4* is X-linked, meaning that in males all neurons carry a single copy of the mutated gene, whereas in females, X-inactivation ensures that neurons exclusively express either the wild-type (WT) or the variant allele. Second, ClC-4 is thought to function primarily within ClC-3/ClC-4 heterodimeric complexes, with homomeric ClC-4 dimers playing a minimal role in its physiological activity.

Therefore, for GoF *CLCN4* variants, a dominant phenotype in patients can be anticipated, as GoF mutations are likely manifested in ClC-3/ClC-4 heteromers, thereby potentially affecting female carriers as well. In fact, the most functionally severe GoF CLCN4 variants identified to date have been observed exclusively in female patients, including I549N and A555V [13]. It is plausible to hypothesize that these severe GoF variants could be embryonically lethal in males.

The interpretation of results for LoF variants in *CLCN4* is more complex. It is well-established that the complete absence of the ClC-4 protein, such as that resulting from early stop codons, leads to relatively mild, non-syndromic disease in males and only mild symptoms in females [10]. In contrast, missense LoF variants in *CLCN4* can cause severe symptoms in both males and females [13]. To account for this dominant phenotype, it has been proposed that missense *CLCN4* LoF variants exert a dominant-negative effect in ClC-3/ClC-4 heteromers [13]. In this study, we developed novel tools and provided the first experimental evidence supporting the dominant effects of *CLCN4* variants in ClC-3/ClC-4 heteromers. We selected four previously investigated, apparently loss-of-function variants, R360S [13], V536M [10], G545S [13] and K560E [13]. The male patient carrying the maternally inherited R360S variant presented with a decrease in social responsiveness and interaction from age 18 months and significant development regression at age 7 years [13]. While clinical data for G545S and K560E remain limited [13], the V536M variant has been identified in both male and female patients exhibiting cerebral atrophy, callosal thinning, and epilepsy, aligning with phenotypes observed for other loss-of-function variants [10,13].

## 2. Materials and Methods

### 2.1. Plasmids

For expression in mammalian cells, we used N-terminally GFP-tagged mouse ClC-3c and N-terminally mCherry-tagged human ClC-4 in the pEGFP vector. The mouse ClC-3 splice variant c differs only in two positions from human ClC-3 with rather conservative changes (I467V, N636S) and has been used in previous studies [12]. For stoichiometric co-expression of ClC-3 and ClC-4, the Encephalomyocarditis virus Internal ribosome entry site (EMCV IRES) sequence was inserted between the open reading frames (ORFs) of GFP-mClC3c and mCherry-hClC4 to form GFP-mClC3c-IRES-mCherry-hClC4-pEGFP bicistronic construct. Single-nucleotide point mutations of hClC4 (hClC4-R360S, -V536M, -G545S, and -K360E) were introduced by PCR and subcloned into the exact position of the hClC4-wt in the IRES construct.

For expression in Xenopus laevis oocytes, the human ClC-4 cDNA or human ClC-4_E281A variant was cloned into the pTLN expression vector [13]. In the ClC-4_E281A background, the selected pathogenic mutants were introduced using standard restriction-free mutagenesis. All constructs were verified by Sanger sequencing.

### 2.2. Cell Culture and Transfection

HEK293 cells and SHSY5Y cells were obtained from the Center of Biological Resources (CRB-HSM) of the IRCSS Hospital San Martino (Genova, Italy) and were grown under the following conditions: Dulbecco’s modified Eagle’s medium-high glucose with 1% glutamine, 1% Sodium pyruvate, 1% penicillin/streptomycin, and 10% fetal bovine serum at 37 °C/5% CO2. All cell culture reagents were purchased from Euroclone (Pero, Italy); transfections were performed using standard lipid based transfection (Effectene; Qiagen, Milan, Italy), as described in [17] using 0.5 µg of DNA for a 35 mm dish, which was split into 4–5 dishes 24 h after transfection.

### 2.3. Expression in Oocytes

RNA was transcribed in vitro using the mMACHINE SP6 Transcription Kit (Thermofisher, Milan, Italy) after linearization of plasmids with MluI.

Adult female Xenopus laevis frogs were obtained from the European Xenopus Resource Center (EXRC) from the University of Portsmouth (UK) and oocytes were harvested and enzymatically defolliculated as described previously [9]. After surgery, frogs were allowed to recover from anesthesia and suitable aftercare was given. Frogs were used no more than two or three times for oocyte harvesting, allowing for at least 2 months of recovery between the operations. The anesthetic used was tricaine (ethyl 3-aminobenzoate methanesulfonate, Merck, Milan, Italy) at a concentration of 1.5 g/L buffered to neutral pH with sodium bicarbonate.

Oocytes were injected with ~6 ng of RNA and incubated at 18 °C for 2–5 days before measurements [12,13].

### 2.4. Whole-Cell Patch Clamp

Whole-cell current recordings were performed on transfected HEK293 cells expressing GFP- and mCherry-tagged ClC-3, ClC-4, and ClC-3/ClC-4 wild-type and mutant proteins, as described in [19]. Briefly, recording pipettes were pulled from borosilicate capillaries (Hilgenberg, Malsfeld, Germany) and filled with a standard intracellular solution containing (in mM) 130 NaCl; 10 Hepes, 2 MgCl_2_, and 2 EGTA, pH 7.3. We used NaCl instead of KCl to reduce contribution of endogenous K^+^ currents. Standard extracellular solution contained (in mM): 160 NaCl, 10 Hepes, 4 MgSO_4_, pH 7.3; pipette resistances ranged between 1.8 and 2.5 MΩ. A 3 M KCl/1% agar bridge served as the ground. Data were recorded using an Axopatch 200 amplifier (Molecular Devices, San Jose, CA, USA), sampled at 100 kHz after filtering at 10 kHz by the amplifier-built-in Bessel filter, and acquired using an NI PCI-6036 interface (National Instruments, Austin, TX, USA) with the GePulse program. To construct current-voltage relationships, the current response at each voltage was averaged over the last third of the corresponding voltage-clamp segment. Data were analyzed using Ana as previously described [17]. Both programs are freely available from http://users.ge.ibf.cnr.it/pusch/programs-mik.htm, accessed on 15 January 2023. Prism (GraphPad, Version 8) was used to perform additional analysis when needed.

### 2.5. Two-Electrode Voltage-Clamp Recording

Two-electrode voltage-clamp recordings of ClC-3 and all ClC-4 variants were conducted in Xenopus oocytes at room temperature (20–22 °C) employing a Turbo TEC_033 amplifier (npi electronic, Tamm, Germany). The standard extracellular solution consisted of 100 mM NaCl, 10 mM Hepes, and 5 mM MgSO_4_ (pH 7.3). All recordings were collected with the GePulse program (version of August 2023) and analyzed using Ana (version of August 2023).

Voltage stimulation protocols followed previously [12,13] and are shown in the Figure insets. To measure ClC-3, ClC-4, and ClC-3/ClC-4 transport currents, 5 ms pulses were applied from a holding potential of 0 mV decreasing in 10 mV steps from 200 to −40 mV. Capacitive and leak currents were corrected using a P/N subtraction procedure, which involved applying a scaled-down measurement protocol (0.2×) and subsequently scaling and subtracting the resulting currents from the raw recordings.

### 2.6. Normalization of Current-Voltage Relationships and Current-Density Relationships

To assess the effects of variants on the voltage dependence of current activation, a specific normalization approach proves particularly informative. The current (or current density) measured in the presence of a given variant at a specific voltage is divided by the corresponding current measured with the correlated wild-type (WT) at the same voltage. In this representation, a variant functionally indistinguishable from WT yields a constant ratio of 1.0 across voltages. A variant that reduces current amplitude without altering voltage dependence will produce a constant ratio < 1.0. In contrast, a variant that affects voltage dependence yields a voltage-dependent ratio. For instance, a variant that shifts activation to more positive voltages will display a ratio with a positive slope. Conversely, gain-of-function variants previously described for *CLCN3* and *CLCN4* in homomeric complexes exhibit ratios with a negative slope [12,13], suggesting destabilization of the inhibitory gating process at less positive voltages. The procedure is illustrated also in Supplementary Figure S1.

### 2.7. Test for the Appearance of Steady Inward Currents at Acidic pH

For the data analysis of currents measured in oocytes at acidic external pH, a specific leak-subtraction was performed, as detailed also in Supplementary Figure S2. First, for each oocyte, currents were measured at pH 7.3 and the resulting current-voltage relationship was fitted in the range −120 mV <= V <= 0 mV with a straight line. The line was extrapolated to all voltages, subtracted from the IVs measured under the various conditions, and normalized to the current at pH 7.3, 160 mV. This is based on the assumption that at pH 7.3, currents for V <= 0 mV are leak.

### 2.8. Fluorescence Confocal Imaging and FLIM-FRET Analysis

Fluorescence confocal and FLIM-FRET measurements were performed on a Stellaris 8 Falcon τ-STED microscope (Leica Microsystems, Mannheim, Germany) equipped with a supercontinuum pulsed (80 MHz) white light laser 440–790 nm and an HC PL APO CS2 100×/1.40 oil immersion objective lens. GFP was excited at 488 nm, and its emission was captured between 502 and 558 nm with a hybrid detector (Leica Microsystems, Mannheim, Germany) in photon counting mode. Lifetime and imaging data were acquired in parallel by scanning the laser with a pixel dwell-time of 3.16 μs/pixel and 16-line accumulation, and a frame size settled to 512  ×  512 pixels. Identical excitation and detection settings were used for conventional GFP imaging. For mCherry, excitation was provided by a 561 nm laser line, and fluorescence was collected from 571 to 628 nm. Bright-field images were obtained using a transmitted-light detector.

Lifetime extraction relied on the fit-free phasor method described in [20]. In this approach, the fluorescence decay at each pixel is transformed into its Fourier components to generate the phasor coordinates G (real component) and S (imaginary component). These values are plotted in the G–S plane as a density map, where the color scale reflects the number of pixels sharing a given pair of coordinates in the image or a selected region of interest. The predominant pixel cluster—identified by Gaussian fitting—was then used to compute the fluorescence lifetime according to:

$$\tau =\frac{S}{2\pi fG}$$

where f = 80 MHz corresponds to the laser pulse frequency. Phasor-based FLIM-FRET analysis was carriedbout using the custom AnaVision software (http://users.ge.ibf.cnr.it/pusch/programs-mik.htm, accessed on 15 February 2024). Regions of interest were restricted to the plasma membrane or to intracellular areas showing intermediate fluorescence signal. FRET efficiency, which depends on donor–acceptor spectral overlap and the relative distance/orientation of the fluorophores, was quantified from donor lifetime reduction, comparing the lifetime in the absence of acceptor (τ_D_) with that measured in its presence (τ_DA_):

$$E=1-\frac{\tau_{DA}}{\tau_{D}}$$

The value of τ_D_ was determined for each experimental session using cells expressing only the donor fluorophore. All fluorescence measurements were obtained from at least three independent transfection experiments.

### 2.9. Statistics

**Figure 1:**
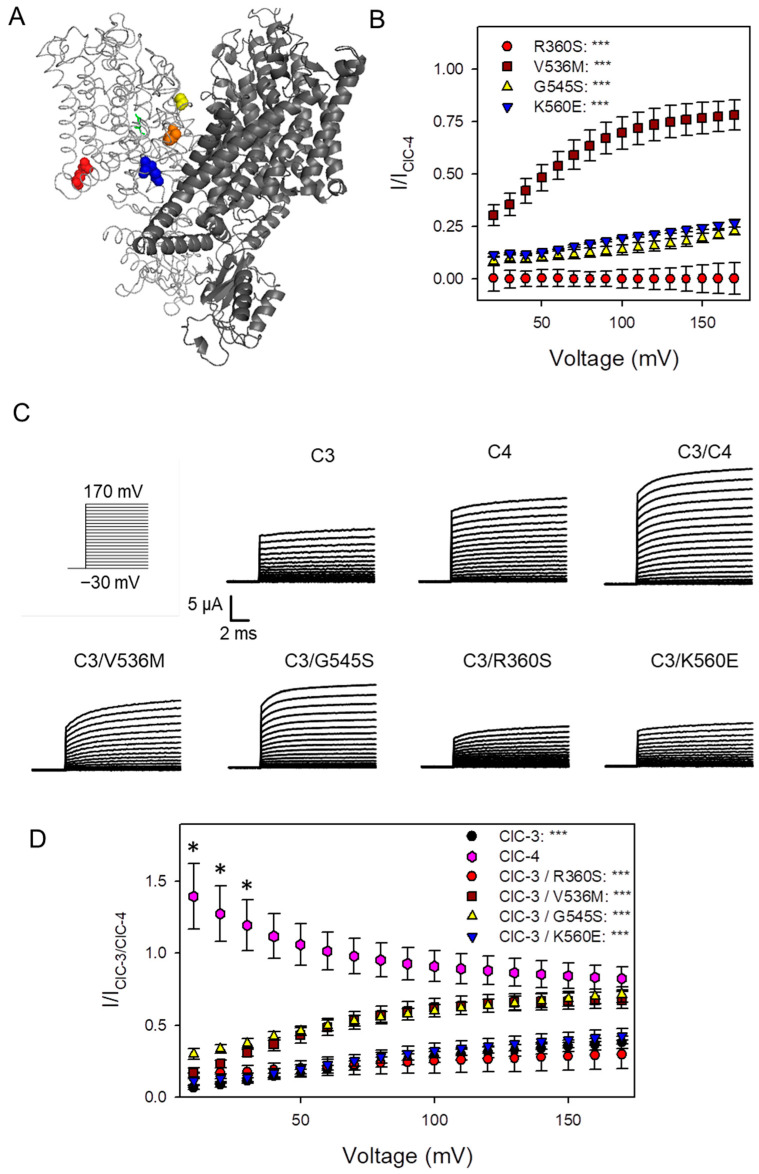
Co-expression of ClC-4 and its mutants with ClC-3 in Xenopus oocytes. (**A**) Three-dimensional homology model of the human ClC-4 protein based on the recently solved structure of the ClC-3 homodimer (Protein Data Bank: 9dnx). The two subunits forming the homodimer are shown in dark and light gray. Mutations are shown in space fill, R360S: red, V536M: orange, G545S: yellow, and K560E: blue. (**B**) Currents mediated by homodimeric ClC-4 variants were normalized to the currents of homodimeric ClC-4 at the same voltage as described in Methods (see also Supplementary Figure S1) (n >= 3 batches for each construct; statistical significance compared to WT ClC-4 injected oocytes is indicated by asterisks: no asterisk: *p* >= 0.05; *: *p* < 0.05; ***: *p* < 0.001). (**C**) Representative recordings of oocytes injected with ClC-3, ClC-4, and with ClC-3 + ClC-4 variants evoked by the voltage-clamp protocol shown on the left. (**D**) Currents of each variant are normalized to the current of ClC-3/ClC-4 at the same voltage. Statistical significance compared to ClC-3/ClC-4 co-injected oocytes is indicated by asterisks (n >= 3 batches of oocytes for each condition with 6–8 oocytes in each batch).

**Figure 2:**
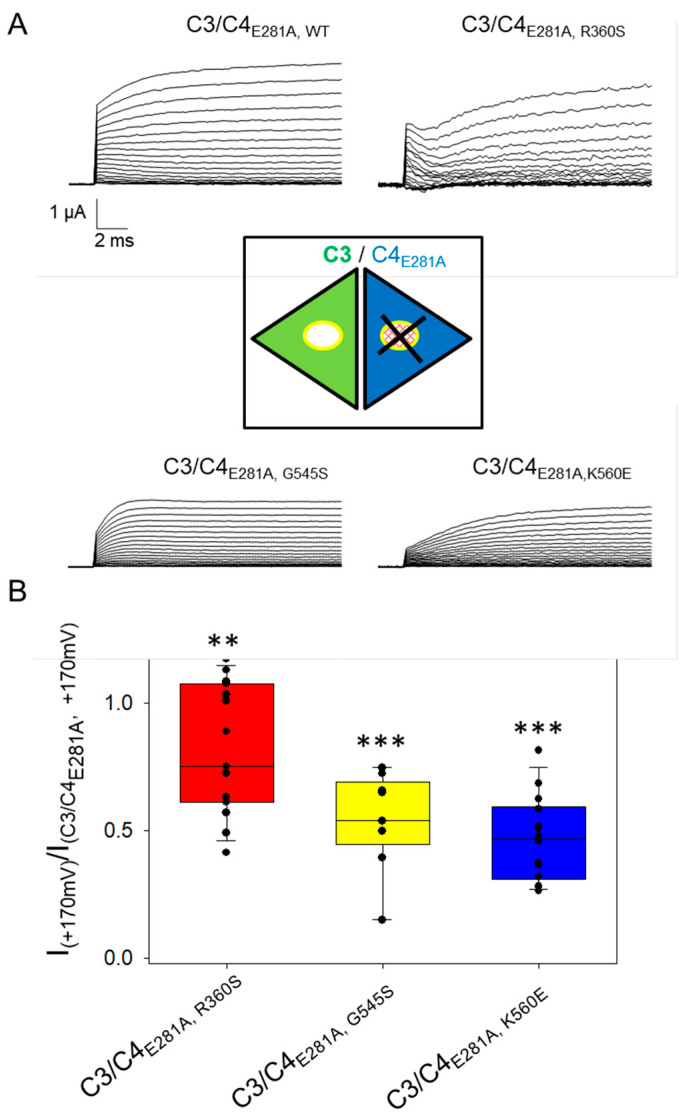
Co-expression of ClC-3 with ClC-4 mutants in the background of ClC-4_E281A in Xenopus oocytes. (**A**) Representative recordings of oocytes co-injected with ClC-3/ClC-4_E281A_ or ClC-3/ClC-4_E281A_ variant, evoked by the same protocol shown in Figure 1. The inset is a schematic cartoon that describes the heterodimer construct formed by ClC-3 (in green) and ClC-4_E281A_, variant (in blue). (**B**) Bar chart of currents measured at +170 mV in ClC-3/ClC-4_E281A_, variant and normalized to those measured in ClC-3/ClC-4E281A, WT expressing oocytes of the same batch. For each construct a total of 3 batches of oocytes with 6–8 experiments for each batch have been analyzed. Asterisks indicate statistical significance when compared to C3/C4 co-injection. Statistical significance is indicated by asterisks: **: *p* < 0.01; ***: *p* < 0.001. Although these results suggest that the *CLCN4* variants act in a dominant-negative manner, the interpretation is complicated by contributions from homomeric ClC-4 currents of unknown proportion. To obtain independent evidence, we employed a strategy previously used for *CLCN7* variants [21]: mutation of the “proton-glutamate” residue (E281 in ClC-4) to alanine abolishes transport currents but preserves dimer assembly [22]. Consequently, any effect of a *CLCN4* variant on the common gating mechanism in heteromeric ClC-3/ClC-4 complexes should also be detectable in the context of the E281A mutation [21]. We therefore introduced the E281A mutation into WT ClC-4 and the variants R360S, G545S, and K560E, and co-expressed these non-transporting constructs with WT ClC-3 (**A**). Currents recorded in these oocytes are exclusively carried by ClC-3 subunits, either in homomeric ClC-3 dimers or heterodimeric ClC-3/ClC-4_E281A,X_ complexes (cartoon in Figure 2), where X represents WT or one of the three variants (see inset in Figure 2). Representative current traces (**A**) qualitatively show reduced currents in oocytes co-expressing ClC-3 with variant-containing E281A constructs compared to those with WT E281A. For quantitative analysis, currents at +170 mV were normalized to the ClC-3/ClC-4_E281A_, WT values within the same batch. As shown in (**B**), a graded reduction was observed, with the strongest effect for K560E, an intermediate effect for G545S, and the least for R360S, consistent with the results in Figure 1. These findings confirm the dominant-negative impact of all three variants in heteromeric ClC-3/ClC-4 complexes.

**Figure 3:**
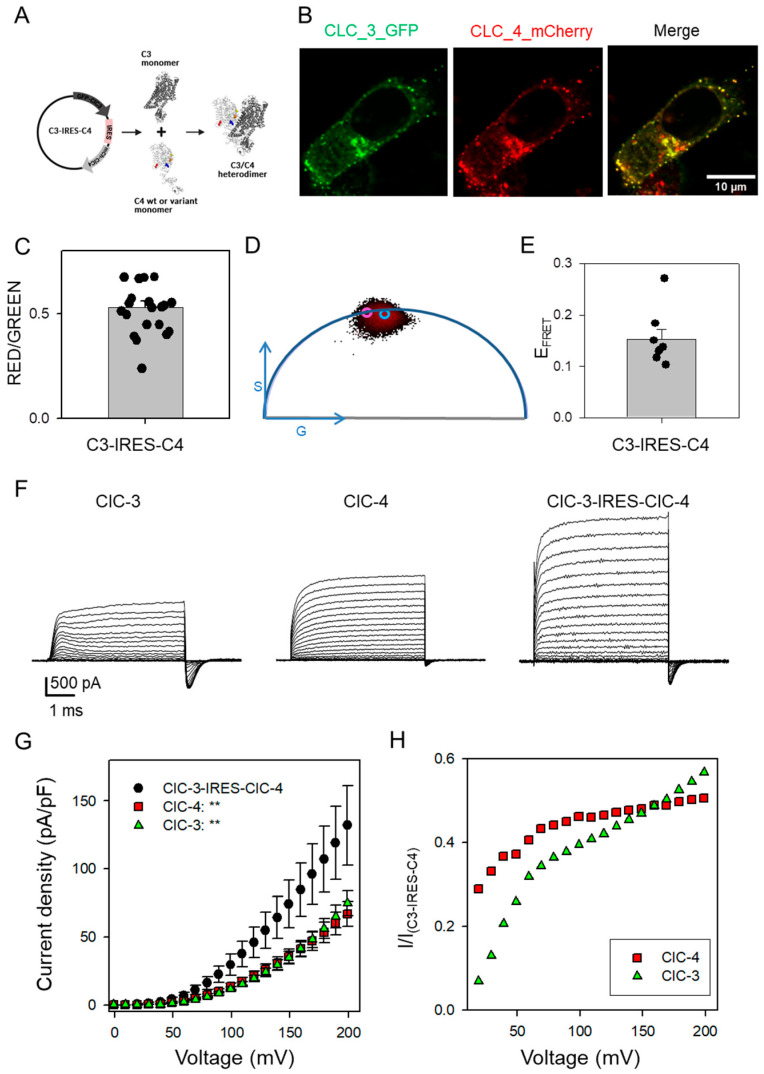
Biophysical phenotype of ClC-3, ClC-4, and ClC-3-IRES-ClC-4 constructs upon expression in mammalian cells. (**A**) Scheme of the C3/C4 IRES construct expressing ClC-3_WT_ and ClC-4_WT_ or variants upon expression in mammalian cell lines. (**B**) Confocal microscopy images of SH-SY5Y cells transfected with the IRES construct expressing N-terminally labeled GFP-ClC-3 (green) and mCherry-ClC-4 (red), along with the merged channel image showing colocalization (image size 40 µm × 40 µm). (**C**) Signal ratio analysis of red and green fluorescence in IRES construct–transfected SH-SY5Y cells indicates that ClC-3 and ClC-4 are expressed at comparable levels. The error bar indicates SD (n = 21 cells). (**D**) FLIM-FRET analysis plot with corresponding lifetime value of ClC-3-GFP co-expressed with mCherry-ClC-4 in the IRES construct. The purple circle indicates the GS-coordinates of the unquenched donor (τ_D_ = 2.22ns), while the blue circle indicates the GS-coordinates in the presence of the acceptor (τ_DA_ = 1.89ns). (**E**) FRET Efficiency analysis on the IRES construct–transfected SH-SY5Y cells. The error bar indicates SD (n = 7 cells). (**F**) Typical ionic currents obtained from ClC-3, ClC-4, and ClC-3-IRES-ClC-4 transfected HEK293 cells. The voltage protocol is the same as shown in Figure 1. (**G**) Current-voltage relationship of steady-state current for ClC-3 (green triangle, n = 16), ClC-4 (red square, n = 7), and ClC-3-IRES-ClC-4 (black circles, n = 13). Statistical significance compared to the ClC-3-IRES-ClC-4 construct is indicated by asterisks for V >= 30 mV (**: *p* < 0.01). For lower voltages, currents were too small to allow for a meaningful comparison. (**H**) A plot of the ratio of the current density of homomeric complexes ClC-3 (green triangle) or ClC-4 (red square) and the corresponding current density measured in ClC-3-IRES-ClC-4 as a function of voltage.

**Figure 4:**
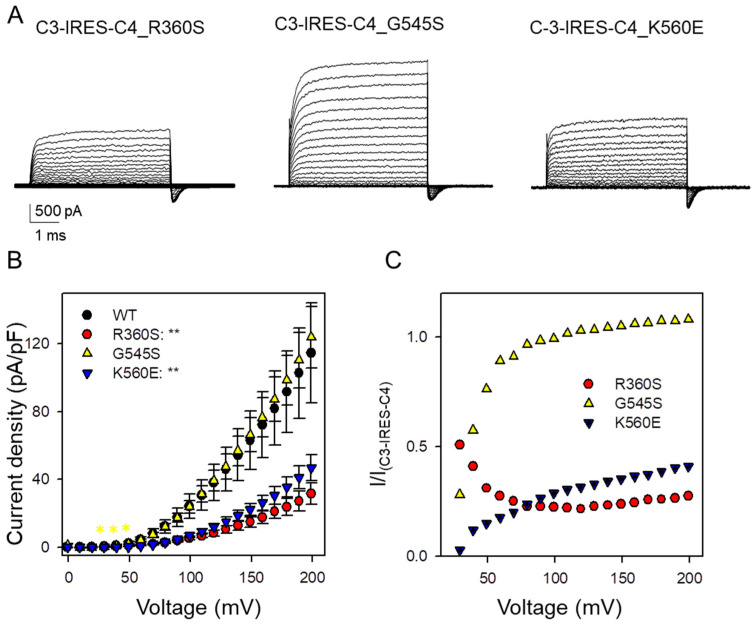
Electrophysiological properties of ClC-3-IRES-ClC-4 construct upon introduction of *CLCN4* variants (R360S, G545S, and K560E) in transfected HEK293 cells. (**A**) Typical currents obtained from ClC-3-IRES-ClC-4variant. The voltage protocol is similar to that shown in Figure 1, with voltages ranging up to 200 mV. (**B**) Current-voltage relationship of steady-state current for ClC-3-IRES-ClC-4WT (black circles, n = 13) and ClC-3-IRES-ClC-4variant (R360S, red circles, n = 10), (G545A yellow triangles, n = 16), (K560E, blue triangles, n = 14). Statistical significance compared to the C3-IRES-C4 construct is indicated by asterisks for V >= 30 mV (*: *p* < 0.05; **: *p* < 0.01). For lower voltages, currents were too small to allow a meaningful comparison. (**C**) Ratio of the current-density of heteromeric ClC3/ClC-4_variant_ (symbols as in (**B**)) and the corresponding current density measured in ClC-3-IRES-ClC-4 WT as a function of voltage.

**Figure 5:**
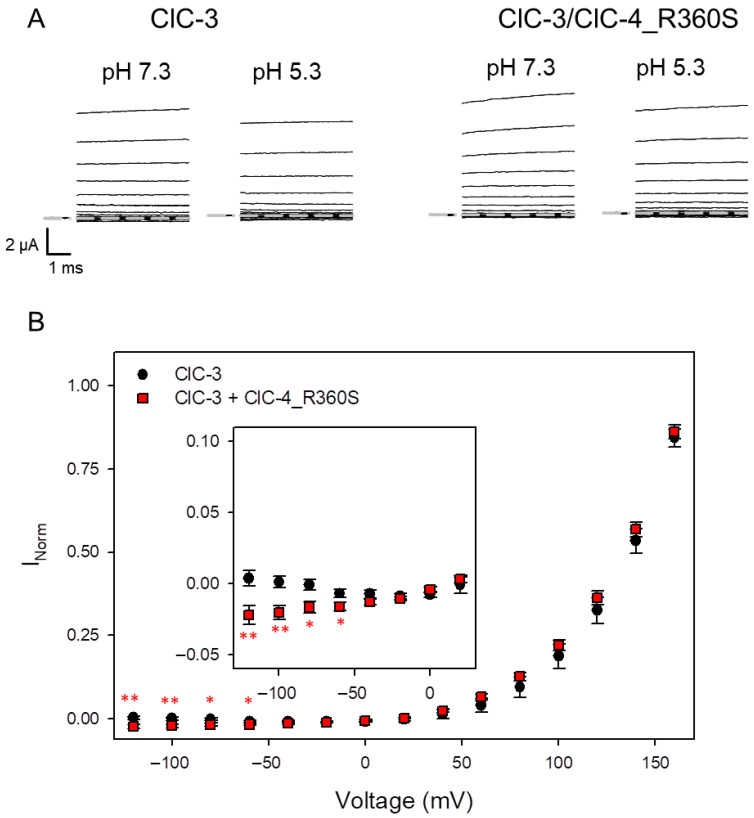
Assay of inward currents at acidic pH of oocytes co-injected with ClC-3 and ClC-4_R360S. (**A**) Typical currents obtained from oocytes at neutral pH and at pH 5.3. Capacitive transients have been blanked for clarity. (**B**) Average normalized current-voltage relationship at pH 5.3 obtained as described in methods (ClC-3: n = 9; ClC-3/R360S: n = 13). The inset shows the data for V <= 20 mV at a different scaling. Statistical significance compared to ClC-3 alone is indicated by asterisks (no asterisk: *p* >= 0.05; *: *p* < 0.05; **: *p* < 0.01).

Data are expressed as mean ± standard deviation (SD) as specified in the Figure legends; n refers to the number of individual cells. Statistical significance was tested by one-way ANOVA followed by Tukey’s post hoc test or Student’s *t*-test as appropriate. For data that were expressed as a ratio of current values compared to a control situation (e.g., Figure 1B,D, Figure 2B and Figure 3H) unpaired Student’s ratio *t*-test was applied, because for each construct different control batches have been employed. For absolute data values (i.e., Figure 3G, Figure 4B and Figure 5) the ANOVA test was used. Asterisks indicate statistical significance as follows: no asterisk: *p* >= 0.05; *: *p* < 0.05; **: *p* < 0.01; ***: *p* < 0.001.

## 3. Results

We employed the consolidated Xenopus oocyte expression system [12,13] to investigate the effects of selected LoF *CLCN4* variants in homo- and heteromeric complexes. Although ClC-4 is predominantly retained in the endoplasmic reticulum under physiological conditions, overexpression in Xenopus oocytes and transfected cells leads to sufficient plasma membrane localization, facilitating robust functional characterization [23].

Variants R360S, V536M, G545S, and K560E, previously described by Palmer et al. [10,13], were selected. Their locations within the protein structure are shown in Figure 1A. Residues V536 (orange), G545 (yellow), and K560 (blue) reside near the dimer interface, whereas R360 (red) is positioned near the intracellular side. As illustrated in Figure 1B, R360S results in the complete loss of function in homodimeric ClC-4 complexes. In contrast, homodimeric V536M, G545S, and K560E complexes exhibit a positive shift in voltage dependence, with currents elicited at more depolarized voltages (Figure 1B) [13]. This conclusion is explained in more detail in Supplementary Figure S1.

We next expressed ClC-3 alone or co-expressed it with either wild-type (WT) ClC-4 or one of the four variants. Representative current traces are shown in Figure 1C. Co-expression of WT ClC-3 with WT ClC-4 produced relatively large outward currents, whereas co-expression of WT ClC-3 with ClC-4 variants resulted in reduced current amplitudes, particularly for R360S and K560E. To assess potential dominant effects, current-voltage (I-V) relationships from each co-expression condition were normalized to those of WT ClC-3 co-expressed with WT ClC-4, representing the physiological condition. Normalization was performed separately for each batch of oocytes, and the resulting ratios are presented in Figure 1D. For variants behaving like WT, the ratio is expected to approximate one. The decreasing ratio for I_ClC-4_/I_ClC-3-ClC-4_ expressed alone (pink diamonds, Figure 1D) at more positive voltages reflects ClC-4′s weaker voltage dependence compared to ClC-3, as ClC-3′s I-V relationship exhibits greater curvature. This is illustrated in Supplementary Figure S3. Notably, variantsV536M and G545S exhibit a voltage-dependent reduction in the ratio at low positive voltages but maintain substantial currents at strong positive voltages, indicating a dominant negative effect on the voltage dependence of heteromeric ClC-3/ClC-4_Variant_ complexes. Variant K560E shows a similar but more pronounced reduction. Variant R360S suppresses currents below those of ClC-3 alone, at least for voltages above 70 mV. For voltages more negative than 70 mV, ClC-3/ClC-4_R360S_ currents were slightly larger than those of ClC-3 alone, even though these differences were not statistically significant. However, the relevance of this observation was further investigated in detail using various approaches (see below). Since V536M and G545S exhibited a rather similar behavior shifting the voltage-dependence to more positive voltages, with G545S exhibiting a stronger effect, we limited the following experiments to variants R360S, G545S and K560E.

To obtain further evidence, we extended our experiments to a mammalian expression system by transfecting HEK293 cells. However, co-transfection of fluorescently tagged ClC-3 and ClC-4 plasmids resulted in highly variable expression ratios between ClC-3 and ClC-4, preventing robust statistical analysis of the functional effects of *CLCN4* variants in heteromeric ClC-3/ClC-4 complexes. Attempts to generate a concatameric ClC-3-GFP-ClC-4 construct to enforce a 1:1 stoichiometry were unsuccessful, likely due to increased toxicity of the construct in bacterial cells. However, we successfully constructed a plasmid encoding GFP-tagged ClC-3 and mCherry-tagged ClC-4 separated by an internal ribosomal entry site (IRES) sequence (Figure 3A). Expression of this IRES construct produced a consistent GFP-to-mCherry fluorescence ratio (Figure 3B,C). Confocal Förster Resonance Energy Transfer measurements using Fluorescence Lifetime Imaging Microscopy (FLIM-FRET) revealed a significant FRET signal between the GFP and mCherry tags, strongly indicating the formation of heteromeric ClC-3/ClC-4 complexes (Figure 3D,E).

Using the patch-clamp technique, we compared currents following transfection with ClC-3, ClC-4, and the ClC-3-IRES-ClC-4 constructs. Representative current traces are shown in Figure 3F. Cells transfected with the ClC-3-IRES-ClC-4 construct exhibited generally larger currents than those transfected with either ClC-3 or ClC-4 alone (Figure 3F,G). In all constructs, capacitive currents were observed upon returning the voltage to 0 mV after the activating voltage steps (Figure 3F) [12,24]. These capacitive currents most likely represent conformational rearrangements of the “gating glutamate” and Cl^−^ ion association/dissociation [25,26]. However, as they are unlikely to have any physiological relevance, we did not analyzed them further [4].

To compare the voltage dependence of heteromeric versus homomeric complexes, we calculated the ratio of current density from homomeric complexes to the corresponding current density measured in ClC-3-IRES-ClC-4 transfected cells at each voltage (Figure 3H). For ClC-3, this ratio exhibited a strong voltage dependence, increasing with more positive voltages (green symbols, Figure 3H). This indicates that the I–V relationship of ClC-3 homomers is more strongly outwardly rectifying than that of heteromeric ClC-3/ClC-4 complexes, reflecting a pronounced effect on the voltage-dependent common gating. Somewhat unexpectedly, compared to ClC-4 homomers, the current–voltage relationship of heteromeric complexes is also less voltage-dependent; specifically, the ratio of currents mediated by ClC-4 homomers to those of ClC-3/ClC-4 heteromers is smaller at voltages below ~100 mV than at more positive voltages (red symbols, Figure 3H). These results suggest that heteromeric ClC-3/ClC-4 complexes possess intrinsically reduced voltage dependence relative to either homomeric form. This reduced voltage sensitivity may be physiologically relevant, potentially enabling more efficient transport at physiological voltages compared to homodimeric complexes.

We next introduced the *CLCN4* variants R360S, G545S, and K560E into the IRES construct and measured currents in transfected cells. Representative current traces are shown in Figure 4A, with average current densities summarized in Figure 4B. Consistent with the oocyte experiments, variants R360S and K560E caused a pronounced dominant reduction in current density. Surprisingly, the current density observed with the G545S variant closely resembled that induced by the IRES construct with WT ClC-4. Further insight was gained by calculating the ratio of current densities at each voltage (Figure 4C). Variant K560E exhibited a clear voltage-dependent reduction, most pronounced at less positive voltages, fully corroborating the Xenopus oocyte results. Also, the G545S variant showed clearly reduced currents at less positive voltage in agreement with the results obtained in Xenopus oocytes.

A rather unexpected finding is that for the R360S variant, the ratio of current density actually increases at less positive voltages (red symbols, Figure 4C). This behavior is more indicative of a gain-of-function effect rather than a loss-of-function [12,13]. Although the variant alone exhibits minimal functional expression at the positive voltages where currents are reliably measured, it appears to enhance the activity of heteromeric complexes at lower voltages.

To explore the possibility that heteromeric ClC-3/ClC-4_R360S transporters exhibit enhanced inward currents at negative voltages at acidic pH, similar to toxic gain-of-function variants of *CLCN3* [12] and *CLCN4* [13], we co-injected the *CLCN4* variant R360S with WT ClC-3 in Xenopus oocytes and measured currents at pH 7.3 and pH 5.3 in the same oocytes (Figure 5A). Subtracting presumed leak currents estimated at pH 7.3 and normalizing currents to that measured at 160 mV at pH 7.3 as described in Methods resulted in normalized IVs shown in Figure 5B. Plotting the same data at an enhanced scale reveals that indeed oocytes co-injected with ClC-3 and ClC-4_R360S exhibit a small but significant increase in inward currents at acidic pH at negative voltages compared to ClC-3 alone (inset in Figure 5B), indicative of a gain-of-function effect.

This observation may help resolve the conundrum reported by Planells-Cases et al. [18], who found a gain-of-function effect of the ClC-3 mutant R418S—equivalent to ClC-4 R360S—demonstrated by resistance to TMEM9-mediated inhibition of vacuole formation.

## 4. Discussion

Using multiple complementary approaches, including direct co-injection in Xenopus oocytes, introduction of variants into a non-transporting mutant background, and patch-clamp analysis in cells transfected with a novel IRES construct encoding tagged ClC-3 and ClC-4, we demonstrate that *CLCN4* variants can exert dominant effects in heterodimeric ClC-3/ClC-4 complexes. This finding is crucial for interpreting the disease phenotypes associated with *CLCN4* missense variants. Specifically, variants V536M, G545S, and K560E, all located near the dimer interface, induce a positive shift in the voltage dependence of activation [13]. This interpretation is based on the assumption that ClC-3 and ClC-4 are governed by a voltage-dependent gating process that saturates at large positive voltages [4]. Thus, the observation that current reduction in these variants compared to WT is stronger at less positive voltages, and is at least partly overcome at more positive voltages suggests that the variants impose a large barrier for gate activation, and therefore a positive shift in the voltage dependence. This interpretation parallels similar findings for disease variants in dominant mutations of the skeletal muscle ClC-1 channel [27].

In contrast, the R360S variant reduces currents in homomeric ClC-4 dimers but increases activity of heteromeric complexes at physiological voltages. This gain-of-function effect was observed in transfected HEK293 cells using the newly developed IRES construct and confirmed in oocytes co-injected with ClC-3. These findings emphasize the importance of employing multiple experimental approaches when studying CLC disease variants. Thus, most likely the disease phenotype associated with this variant arises from a toxic gain-of-function effect. Notably, the R360S variant was identified in a male patient with severe clinical symptoms but no epilepsy [13].

In conclusion the methods developed here will be valuable for future studies of *CLCN4* and *CLCN3* variants exhibiting qualitatively novel functional phenotypes, especially in the context of heteromeric ClC-3/ClC-4 complexes.

## Data Availability

Primary data shown in the Figures will be made available upon reasonable request.

## Supplementary Materials

The following supporting information can be downloaded at: https://www.mdpi.com/article/10.3390/cells14241973/s1.
