# Supplementary material for: Dominant Action of CLCN4 Neurodevelopmental Disease Variants in Heteromeric Endosomal ClC-3/ClC-4 Transporters
Source: Cells. 2025 Dec 11;14(24):1973. doi: 10.3390/cells14241973 (PMC12731782; doi:10.3390/cells14241973)
Supplement: Supplementary file 1 [file cells-14-01973-s001.zip › cells-4006094-supplementary.pdf]

Supplementary Figures

Supplementary Figure S1

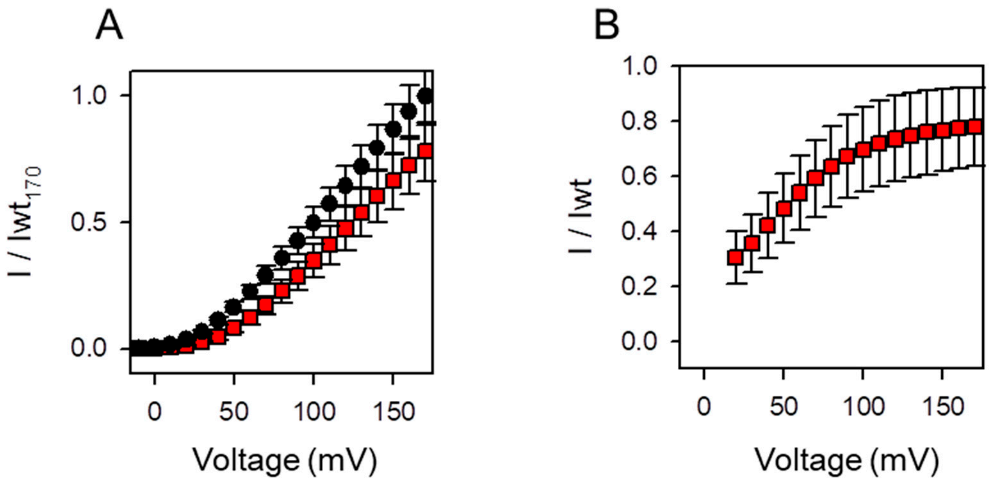

Supplementary Figure S1. Normalization of current-voltage relationships. Panel A shows average current voltage relationships obtained from several batches of oocytes, injected with WT ClC-4 (black circles) and the V536M (red squares) disease causing variant, where for each batch currents were normalized to the mean value measured for WT injected oocytes at 170 mV. In panel B, that shows the same values for this variant as Fig. 1B, the values obtained for the mutant in panel A are divided by the values obtained for WT at the same voltage, i.e. the values corresponding to the red symbols divided by the values corresponding to the black symbols of panel A. A variant that is identical to WT would result in a constant value around 1 with this normalization. A variant that shows a voltage-dependent ratio with increasing values towards more positive voltages is interpreted to cause a shift of the voltage-dependence to more positive voltages. This behavior is much more difficult to detect by just inspecting the I-V relationship shown in panel A.

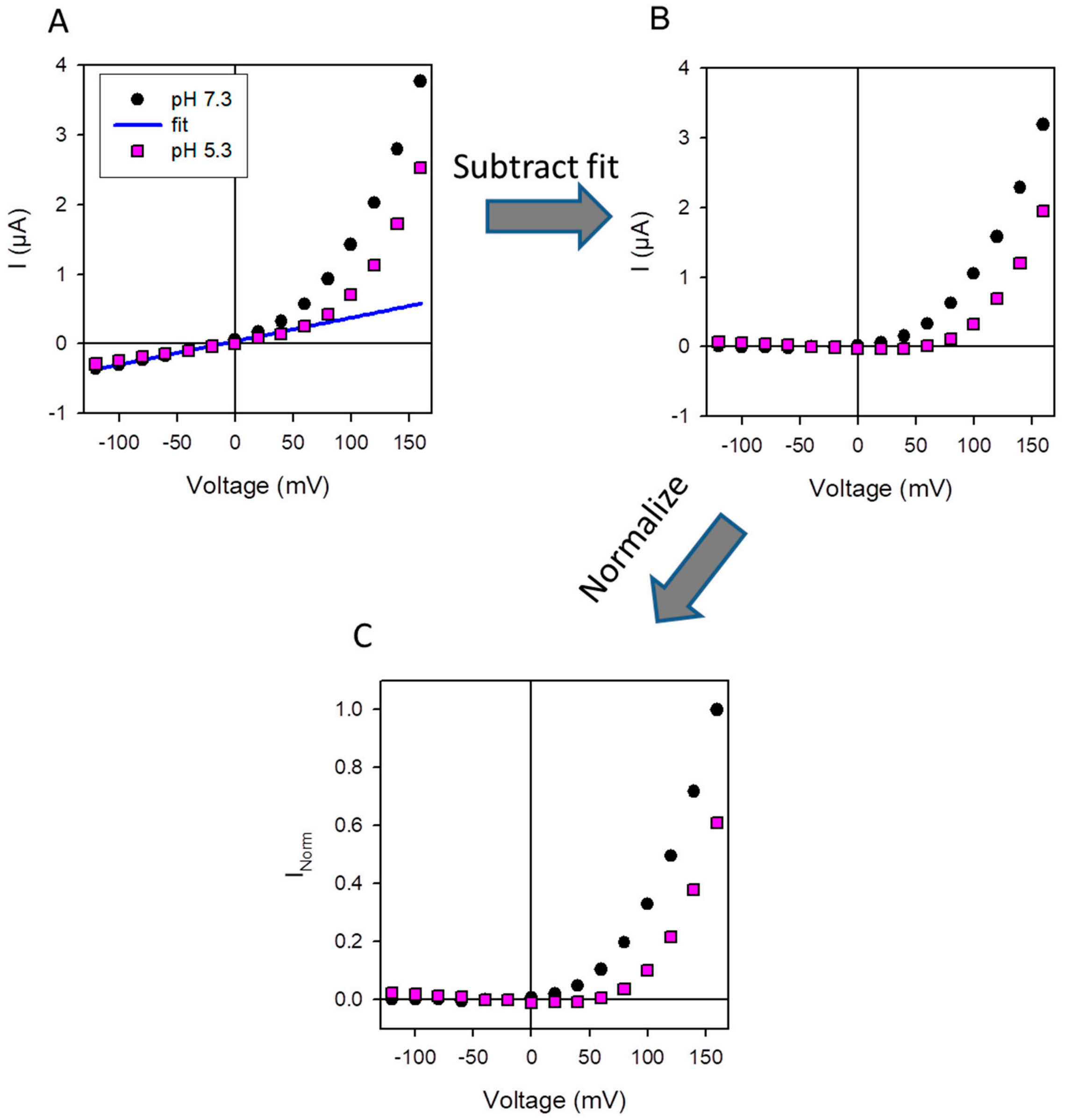

Supplementary Figure S2. Test for the appearance of steady inward currents at acidic pH. The procedure is illustrated for an exemplary ClC-3 WT injected oocyte. Panel A shows currents measured at pH 7.3 as well as at pH 5.3 from the same oocyte. Currents at pH 7.3 for V<=0 mV were fitted by a straight line (blue line). The underlying assumption is that the currents at negative voltages are mostly leak / endogenous currents unrelated to ClC-3. This line was extrapolated to positive voltages and subtracted from all data points resulting in panel B. Then, all leak-subtracted data points were divided by the (leak subtracted) current measured at 160 mV at pH 7.3, resulting in panel C. These normalized values were averaged over several oocytes.

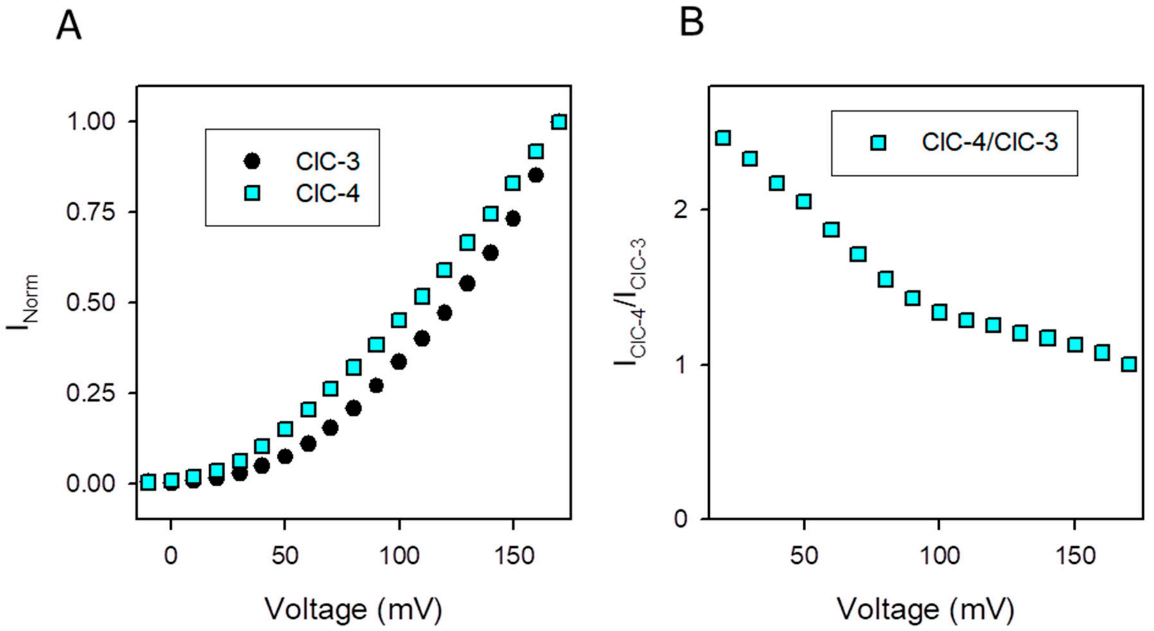

Supplementary Figure S3. More pronounced rectification of ClC-3 vs. ClC-4. Panel A shows the average of normalized current-voltage relationships for ClC-3 and ClC-4 injected oocytes, respectively. Values were normalized to the current measured at 170 mV. Panel B shows the ratio of the normalized currents measured for ClC-4 and those measured for ClC-3. The more pronounced rectification of ClC-3 compared to ClC-4 becomes evident in this representation.
